# Optimal clinical management of tenosynovial giant cell tumours: a UK perspective

**DOI:** 10.1302/2633-1462.74.BJO-2025-0365.R1

**Published:** 2026-04-03

**Authors:** The TGCT UK Consortium

**Affiliations:** 1 TGCT Support, Wayne, New Jersey, USA; 2 Leicester Orthopaedics, University Hospitals of Leicester NHS Trust & Leicester Cancer Research Centre, Leicester, UK; 3 Orthopaedic Oncology, Royal National Orthopaedic Hospital, Stanmore, UK; 4 Liverpool University Hospitals NHS Foundation Trust, Liverpool, UK; 5 Sarcoma Specialist centre, Sarcoma, UK; 6 TGCT Support, Edgware, UK; 7 Sarcoma Unit, University College London Hospital NHS Foundation Trust, London, UK; 8 Sarcoma Unit, Royal Marsden Hospital and Institute of Cancer Research, London, UK; 9 Royal Orthopaedic Hospital, Birmingham, UK; 10 Oxford Cancer and Haematology Centre, Churchill Hospital, Oxford University Hospitals NHS Foundation Trust, Oxford, UK; 11 St James's Institute of Oncology, Leeds, UK; 12 Leeds Institute of Medical Research, University of Leeds and Leeds Teaching Hospitals National Health Service Trust, Leeds, UK; 13 The Robert Jones and Agnes Hunt Orthopedic Hospital, Oswestry, UK; 14 The Life Raft Group, Wayne, New Jersey, USA

**Keywords:** Sarcoma, Oncology, PVNS, TGCT, Orthopaedics, Tenosynovial giant cell tumours, sarcoma, giant cell tumour, medical oncology, Delphi methodology, systemic therapies, lesions, joint arthroplasty, morbidity, MRI scanning

## Abstract

**Aims:**

Tenosynovial giant cell tumour (TGCT) management is variable across the UK. Our aim was to examine these differences in clinical practice and develop consensus statements regarding the management of TGCTs.

**Methods:**

A three-stage modified Delphi technique was conducted with surgical, clinical, and medical oncology experts from across the UK. Key areas of controversy were identified in a virtual meeting on 23 January 2025. This was followed by an online questionnaire that was iteratively refined, and a virtual consensus meeting on 2 September 2025, to discuss topics where agreement had not yet been reached.

**Results:**

This consensus developed a definition for unresectable TGCT and criteria for patient referral from TGCT local management to multidisciplinary team (MDT) review with access to current appropriate treatment options and further defined unresectable TGCT. Diffuse, recurrent, and/or unresectable TGCT, or those requiring complex procedures should be reviewed through a centralized MDT case review at a sarcoma centre, given the complex multidisciplinary nature of TGCT management.

**Conclusion:**

The clarification of classification of localized and diffuse TGCTs, and the definition of unresectable TGCTs, as well as standardizing the criteria for referral to a sarcoma MDT, will facilitate the improved management of TGCTs across the UK and address regional resource challenges.

Cite this article: *Bone Jt Open* 2026;7(4):482–490.

## Introduction

Tenosynovial giant cell tumours (TGCTs), formerly known as pigmented villonodular synovitis (PVNS) or giant cell tumour of the tendon sheath (GCT-TS), is a rare, locally aggressive mesenchymal neoplasm arising from the synovium of a joint, tendon sheath, or bursa.^1,2^ TGCTs affects a single joint with location varying by subtype.^3^ TGCT is molecularly characterised by a colony stimulating factor 1 (CSF1) gene aberration leading to cellular proliferation. The diffuse (D-TGCT) and localized (L-TGCT) subtypes share this molecular aetiology but differ clinically. D-TGCT has higher disease burden and local recurrence rates (LRRs), whereas L-TGCT presents more commonly with lower volume disease, lower LRRs, and more favourable surgical outcomes.^4,5^

Although rarely life-threatening, TGCT and its treatment can significantly impact patients’ quality of life and function. Despite efforts to harmonize the management of TGCT globally,^6^ nomenclature variation, lack of centralized specialist review processes and referral pathways, lack of consensus on best practices, and inconsistent follow-up schedules remain barriers. Non-surgical options, such as off-label systemic therapies (e.g. imatinib, nilotinib), are available; however, lack of reimbursement and restrictions have led to inconsistent treatment strategies across regions, hospitals, and physicians.

Thus, a consensus was developed among orthopaedic consultants, medical/clinical oncologists, patient representative, clinical sarcoma nurse, and patient advocacy groups (TGCT Support and Sarcoma UK) with the aim of harmonizing the management of TGCTs across the UK based on best practices in real-world clinic. This type of consensus has precedent in other diseases such as chondrosarcoma.^7^

**Fig. 1:**
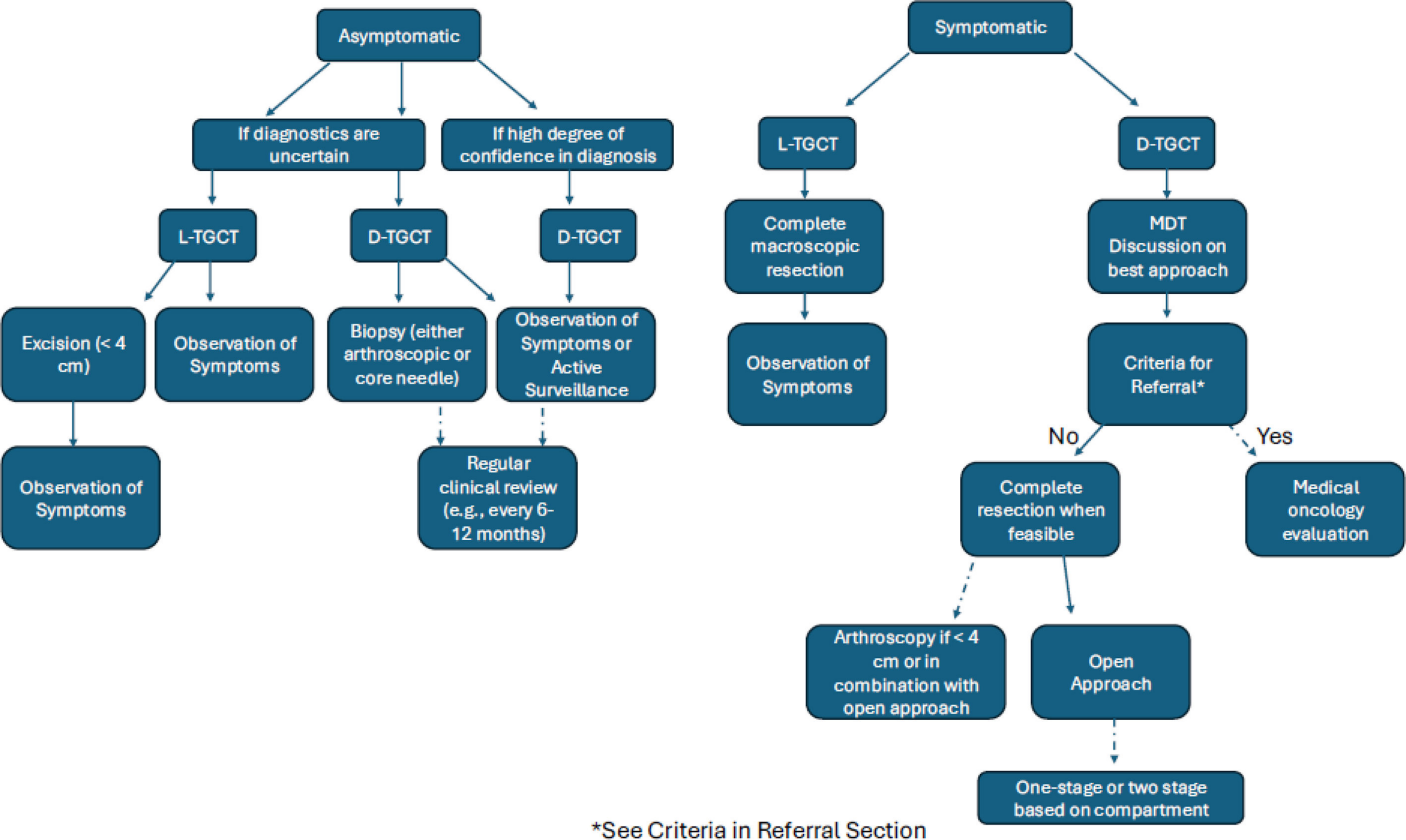
Proposed treatment algorithm for the management of tenosynovial giant cell tumours.

Due to the lack of formal NHS referral pathway for TGCT patients to sarcoma specialists, the unpredictable disease course, and the high burden of disease and LRR for patients with D-TGCT, we sought to: 1) determine criteria for referral of patients from general practitioners and regional non-specialist centres to tertiary sarcoma centres for multidisciplinary assessment and treatment, such as role of surgery, systemic therapies, radiotherapy, and clinical trials; and 2) define best practices among the consulted experts with considerations of regional resources. In addition, we propose a referral and treatment paradigm in Figure 1.

## Methods

A systematic literature search was conducted and in combination with UK key opinion leaders, the group reached consensus on key aspects of the management of TGCTs with considerations of the NHS structure. Key opinion leaders were chosen based on the most frequently consulted specialists in the TGCT Support Registry, ensuring broad geographical coverage, real-world expertise, and representation from regional centres and experts in orthopaedics and medical/clinical oncology. Overall, 21 experts from independent institutions were invited, and 20 accepted. The group included six orthopaedic surgeons and orthopaedic oncologists, four medical oncologists, two clinical/medical oncologists, one acute pain nurse specialist, one clinical sarcoma nurse, and three physiotherapists, as well as one patient representative and two patient advocacy organizations.

A modified Delphi method was used comprising a three-stage process of a pre-meeting of expert consultants, an online questionnaire, and a consensus meeting to address misalignment. Differences in clinical approaches were discussed in a virtual meeting on 23 January 2025, followed by a questionnaire capturing consultants’ standard practices (Supplementary Material table i). The online questionnaire was modified in several iterations following anonymous feedback and refinement from experts. One-on-one outreach was conducted to ensure full participation; thus, the facilitator was not blinded. Statements were modified based on feedback until consensus was reached. The final consensus meeting was held on 2 September 2025, to resolve remaining discrepancies and assess the statements for further refinement. This was considered a modified Delphi method due to the non-anonymous elements such as 1:1 outreach and alignment meeting. All but two members attended the alignment meeting, and the two absent members provided feedback at a later date.

Levels of evidence and consensus was determined based on responses from the survey. Domain-specific statements on surgical techniques and systemic therapy candidates were addressed by orthopaedic and medical oncology experts, respectively. In the virtual meetings, all experts were encouraged to discuss and engage on the various statements regardless of speciality; however, consensus was reached by domain-specific experts. General statements regarding referrals or surveillance were addressed by the whole group. Consensus was evaluated as strong (> 75% of participants/experts agreeing with a statement), moderate (50 to 75% of participants/experts agreeing with a statement), or low (< 50% agreement with a statement/no clear agreement).

## Results

### TGCT nomenclature

In 2013, the World Health Organization unified multiple terms such a nodular tenosynovitis, giant cell tumour of the tendon sheath, and PVNS under a single entity, classifying TGCT as a locally aggressive intermediate neoplasm.^8^ This reclassification reflects advancements in the pathogenic drivers, neoplastic process, and limited response to steroids and anti-inflammatory treatments. However, terminology remains inconsistent, reflecting an ongoing transition from PVNS to TGCT. While TGCT is the preferred nomenclature in medical and surgical oncology, PVNS continues to appear in radiology reports, referral letters, and among some orthopaedic surgeons, highlighting a need for greater harmonization and clarity across specialties.

### Diffuse versus localized TGCT characterization

Subtyping of TGCT should be determined by radiological appearance, rather than histopathology (consensus level: strong). Microscopically, there are few distinctions; therefore, subtypes are dictated by radiological findings. L-TGCT (or nodular used interchangeably) is described as well-circumscribed, or encapsulated, typically confined to one site or one compartment within a joint. This subtype can present as a single lesion or multiple lesions, as long as each is localized and well-defined.^6^ L-TGCT is often intra-articular.

The classification of well-defined, lobulated lesions in multiple joint compartments (e.g. suprapatellar pouch and posterior cruciate ligament) as L- or D-TGCT differed between experts. To align with international consensus, any well-circumscribed, non-infiltrative focal mass(es) is classified as L-TGCT (consensus level: moderate). An “intermediate” or “multifocal localized” category for multi-compartmental well-defined lesions lacking infiltrative features was proposed (consensus level: low). Multifocal L-TGCT was considered surgically manageable with outcomes comparable to L-TGCT after complete resection (consensus level: moderate). The patient representative cautioned that an additional category may complicate classification without clear benefit and should remain provisional pending further evidence.

D-TGCT exhibits an infiltrative, multifocal growth pattern that may involve multiple joint compartments, with irregular margins, extensive joint involvement, and possible extra-articular extension (consensus level: strong). Imaging findings may include blooming artifact on gradient echo (GE) MRI, joint effusion, bone erosions, and subchondral cysts, particularly in joints with tight capsules (such as the hip). Extra-articular disease may extend into periarticular soft tissues, muscles, and neurovascular or tendon structures. Notably, lesion number and size alone are insufficient for diagnosing D-TGCT (consensus level: strong).

### Diagnosis

Diagnostic imaging: MRI is the optimal imaging modality for diagnostic identification and characterization of TGCT. MRI should be recommended as part of the standard diagnostic work-up (consensus level: strong). Conventional radiography has limited value but may identify calcifications, bone erosions, subchondral cysts, and joint degeneration, particularly in in longstanding or advanced disease (consensus level: strong).

The importance of contrast agents (e.g. gadolinium) as well as fluid-sensitive sequences (e.g. STIR or T2 FS) was highlighted for detecting inflammation, differentiating tissue planes, and identifying haemosiderin. However, these agents may not be necessary and may be resource dependent (as non-contrast scans free up local imaging capacity) (consensus level: low).

Aligned with global recommendations from an international consensus for the management TGCT,^6^ a minimal MRI protocol recommendation should include T1-weighted, T2-weighted and a fluid-sensitive sequences (consensus level: strong). Subtraction imaging (pre- and post-contrast T1) was suggested to improve lesion delineation. Spectral fat suppression is preferred over inversion recovery unless metal artifact is present.

Biopsy: Approaches to confirming radiological diagnoses of L-TGCT and D-TGCT are nuanced, and opinions varied whether a core-needle biopsy is required for diagnosis. For TGCTs where high-quality MRI are interpreted by musculoskeletal radiologists with extensive experience of assessing TGCT and the MRI is highly suggestive (e.g. suspected D-TGCT displaying blooming artifacts), core-needle biopsy was deemed unnecessary (consensus level: strong). For small, localized lesions, en bloc excisional biopsies could proceed directly if the radiological features are consistent with that of TGCT (consensus level: moderate). For localized lesions, tissue-confirmed diagnosis via core-needle biopsy before excision may be appropriate in some cases where imaging is inconsistent with TGCT after review in a sarcoma MDT (consensus level: low). Technical and feasibility challenges may arise when performing core-needle biopsy on localized lesions < 2 cm. All removed surgical specimen should be sent for histopathological confirmation of diagnosis and resection margins. If the radiological characteristics are atypical, then the case should be referred to the local sarcoma centre prior to any surgery as per national UK guidelines to rule out a differential diagnosis of sarcoma (consensus level: strong).

Core-needle biopsy (14 to 16 gauge) is warranted in cases where the diagnosis is uncertain, clinical, and radiological appearances are atypical, lesions are in anatomically challenging or high-morbidity locations, or diagnosis is essential to formulate an appropriate treatment plan (consensus level: strong). Core-needle biopsy could be considered in suspected D-TGCT before undertaking major interventions such as extensive surgery and is essential before systemic therapy (consensus level: moderate). Biopsies for large, diffuse, recurrent TGCT or atypical radiological appearance should be reviewed at a sarcoma centre and/or with experienced musculoskeletal histopathologists (consensus level: strong).

### Treatment

Patients with suspected D-TGCT, TGCT in atypical locations, or those requiring complex procedures be reviewed through a centralized MDT case review at a sarcoma centre, given the multidisciplinary nature of TGCT management (consensus level: strong).

### Surgery

Asymptomatic localized TGCT: The primary approach for asymptomatic L-TGCT should not be surgical (consensus level: strong). Experts cautioned against intervening surgically when patients are asymptomatic, due to the potential morbidity of a surgical procedure. Surgery should only be considered if the lesion becomes symptomatic and/or impacts physical function.

Active surveillance is recommended in the absence of symptoms or functional impairment (consensus level: strong). Surveillance may involve clinical follow-up and/or interval imaging as clinically indicated every six to 12 months. Active surveillance strategies should be discussed with patients and may vary by extent of disease or location, lesion characteristics, and patient-specific factors. In some instances, small lesions (e.g. < 4 cm) may undergo excision when the lesion is easily accessible and surgical removal is low risk (consensus level: low).

Symptomatic localized TGCT: Excisional biopsy or macroscopic complete resection should be universally recommended for symptomatic L-TGCT (consensus level: strong). For L-TGCT < 4 cm, excision by arthroscopy may be performed if macroscopic complete resection is achievable. Symptom burden should be the key driver for intervention.

Experts preferred open surgical approaches over arthroscopy for reliable, complete resection, especially for large lesions (e.g. ≥ 4 cm) and dependent on joint compartment involved (consensus level: strong). The use of arthroscopic resection could be appropriate for lesions that can be removed en bloc without contamination of the joint at easily accessible joint locations, particularly in anterior compartments or in specific joints (e.g. knee or ankle) (consensus level: moderate).^9,10^ Overall, an open approach was recommended due to concerns about piecemeal removal during arthroscopy (consensus level: moderate). Posterior arthroscopies of the knee and ankle should not be advised due to technical difficulties achieving complete macroscopic resection and concerns with contaminating the joint (consensus level: strong).

Experts emphasized that morbidity varies by site, symptom management with non-invasive approaches, and surgical decisions should balance achieving complete removal with preservation of function.

Asymptomatic diffuse TGCT: The primary approach for asymptomatic D-TGCT should be active surveillance with regular clinical follow-up to assess disease progression and/or symptom development (consensus level: moderate). Treatment decisions should be guided by the presence and progression of symptoms, rather than imaging alone.

Management should involve detailed discussions with patients about the chronic nature of D-TGCT, the high LRR after surgery, risks and morbidity associated with complex or staged surgeries. Patients should be encouraged to participate in management decisions, balancing risks of surgery and subsequent recovery, with active surveillance, or medical treatment (consensus level: strong). MDT (or tumour board) discussions provide value to evaluate surgical, medical, and surveillance options based on individual patient factors. Treatment delays in asymptomatic complex patients with anterior and posterior compartment involvement of the knee may be followed by higher morbidity, staged procedures in the future (consensus level: low).

If complete macroscopic resection of D-TGCT is not feasible, debulking is the likely surgical alternative. If symptoms develop and suboptimal surgical outcomes are expected, then a referral to sarcoma medical/clinical oncology could be considerations of primary systemic therapy (consensus level: moderate).

### Symptomatic diffuse TGCT

Symptomatic D-TGCT should be managed by a multidisciplinary sarcoma centre and MDT review and discussion, including surgical, medical/clinical oncology, and radiology teams, should be recommended for treatment planning (consensus level: strong). Multidisciplinary discussion across specialties helps balance disease burden, surgical feasibility, risk of morbidity, and the role of systemic therapy.

Complete macroscopic resection via open vision is the preferred approach if achievable with acceptable morbidity and preservation of function (consensus level: strong). If complete resection is not feasible, partial resection (debulking) surgery of the symptomatic portion of the disease (e.g. anterior compartment of knee) may proceed only if recommended by a sarcoma MDT review. Experts agreed that debulking is a feasible but often suboptimal option that is typically performed due to lack of better alternatives and this should be discussed with a sarcoma MDT (consensus level: moderate). Debulking was viewed as a strategy to achieve symptom control, though the degree of symptomatic benefit achieved was acknowledged to be variable. The patient representative highlighted the importance of clarifying the role and expected results of debulking to patients for informed decision-making. Systemic therapy may be preferred following MDT discussion and patient preference (consensus level: moderate).

Arthroscopic surgery was not favored for D-TGCT due to the risk of widespread disease dissemination (consensus level: strong). In narrowly defined exceptions, arthroscopic resection may be performed, such as when the disease is anterior and intra-capsular, or within a combined arthroscopic anterior and open posterior approach. When performed, arthroscopic resection requires substantial experience and expertise.

Referral for consideration of systemic therapies is recommended for discussion of alternatives for patients with recurrent TGCT, or D-TGCT or L-TGCT in complex locations (consensus level: moderate).

### Unresectable TGCT

Clinical judgement must consider risk versus benefit, wider multidisciplinary treatment options, and patient quality of life, if planning any large and morbid surgeries. Defining unresectability is usually a collaborative decision between surgeons and radiologists based on imaging and anatomical challenges (e.g. nerve/tendon involvement).

The current international consensus suggests that D-TGCT with nerve entrapment/tendon/blood vessel involvement should be considered unresectable.^6^ Further criteria for unresectability are proposed as follows: 1) anatomically complex involvement (e.g. nerve entrapment or complex multifocal TGCT); 2) high surgical morbidity/risk (e.g. long recovery or potential complications such as stiffness, arthrosis, or lack of benefit post-surgery); 3) no symptomatic benefit anticipated; and 4) surgical technique possible but morbid (e.g. technically feasible does not always mean appropriate, patient impact matters).

Overall, the determination of ‘unresectable’ is a contextual, patient-specific judgment balancing technical feasibility with functional outcomes and risk. All complex cases should be discussed with a sarcoma MDT review, to include the views of other surgeons, radiologists, and medical/clinical oncologists in determining the appropriateness of surgery for a patient (consensus level: moderate).

### Joint arthroplasties in TGCT

Arthroplasty (joint reconstruction or arthroplasty) may be appropriate in TGCT patients, but only when secondary degenerative joint disease (e.g. osteoarthritis or joint erosion) is advanced and mobility is not preserved (consensus level: strong). Arthroplasty should not be used as a treatment for TGCT directly and does not eradicate TGCT, reduce recurrence risks or effectively manage symptoms of TGCT without the presence of arthrosis. Although, arthroplasty may be successful to manage symptomatic joint damage with functional consequences caused by chronic disease progression. If a joint arthroplasty is planned, it should be noted that one- or two-stage synovectomy is still necessary to resect TGCT.

### Radiotherapy

Radiation as a treatment for TGCT should be limited to very select patients after sarcoma MDT review and only once all other options are exhausted (consensus level: strong). Concerns regarding its use include secondary radiation-associated malignancies, iatrogenic arthrosis, increased risk of surgical sequelae complicating joint arthroplasty, hemarthrosis, soft-tissue fibrosis, and joint stiffness limiting its utility.

### Systemic therapies

Oncology referrals for systemic therapy: Medical oncology referral is usually not necessary in the initial treatment settings, but is recommended early in cases of symptomatic, diffuse, recurrent, or unresectable TGCT, or when surgery is associated with partial resection and high morbidity, or when surgical options are exhausted, carry a high risk of local recurrence is associated, or involve difficult surgical locations (e.g. posterior capsule or ankle) (consensus level: moderate). Symptomatic recurrence may be a common trigger for medical oncology involvement (consensus level: low).

The decision to refer to medical oncology is made on a case-by-case basis within an MDT setting (consensus level: strong). Incorporating patient preference, especially when symptoms persist, or following progression/recurrence after prior surgeries was emphasized. Additionally, several surgical experts suggested a proactive approach, referring for oncology review early in the management plan process if TGCT is diffuse, only partially resectable, recurrent, or in difficult surgical locations.

We propose the following criteria as indication for referral for oncology review: 1) symptomatic biopsy-proven TGCT, and one or more of the following: 2) symptomatic D-TGCT; or 3) unresectable TGCT (e.g. high risk of surgical morbidity); 4) TGCT not amenable to complete surgical resection; 5) recurrence after prior surgery without maintained function or disease control; or 6) patient preference. All experts agreed that prior surgery is not a strict requirement before initiating systemic therapy in patients with symptomatic and D-TGCT.

Several key challenges to implementation of the referral criteria include situations where sarcoma oncologists in some regions do not manage TGCT, as it is a non-malignant disease, and that sarcoma oncology services are stretched in some regions which results in prioritization of malignant diagnoses. Furthermore, access to imatinib (the only available TGCT systemic therapy in the UK at the time of the consensus meeting) is regionally restricted according to individual hospital policies, or only permitted in specific clinical settings (e.g. neoadjuvant prior to scheduled surgery).

Neoadjuvant systemic therapies: There is a potential role for neoadjuvant therapy, especially in cases of bulky disease or when systemic therapy may improve surgical outcomes (consensus level: moderate). Some systemic therapies, most commonly imatinib, may be used in this setting particularly where policy or funding dictates that systemic therapy is tied to surgical planning. Patients may later decline planned surgery due to symptomatic and functional improvement achieved by systemic therapy alone.

Systemic therapy availability: There are no licensed systemic therapies for the treatment of TGCT available in the UK. Imatinib is available for off-label use in some regions, although availability and clinical indications vary between regions and are determined by individual hospital drugs and therapeutics committees, such that there is considerable variability of access across the country. Imatinib is the standard-of-care systemic therapy option in the UK at present (consensus level: strong). However, clinical trials should be the preferred option when available (consensus level: strong).

Patients prioritized for systemic therapies usually have significant symptom burden and/or D-TGCT that is unlikely to be cured with surgery. Discussions with patients regarding risks, benefits, tolerability, and toxicity of systemic therapies are vitally important to informed consent, especially with newer agents. Future fertility, family planning, and pregnancy considerations should be discussed with younger patients. Experts emphasized the importance of individualized treatment planning and open dialogue with patients.

### Surveillance and follow-up

Experts defer to radiologist-defined protocols, especially in experienced sarcoma centres, emphasizing tailored imaging based on the tumour and clinical context. individualized MRI monitoring schedules may be developed in shared decision-making with the patient, especially when a patient has specific concerns (consensus level: low). Patients may feel more reassured and less anxious when their TGCT is tracked with routine imaging and may take time to accept symptomatic-only monitoring.

Clinical symptoms and physical function should guide post-operation decision-making and imaging (consensus level: strong). postoperative baseline imaging was recommended for patients with D-TGCT to assess residual disease (consensus level: moderate) and should be performed within six months post-surgery. If contrast agents are to be intermittently used, pre-surgical baseline scan and initial postoperative follow-up are most valuable for planning care.

Follow-up clinical symptom assessment should be conducted at six and 12 months post-operation (consensus level: strong). Experts often assumed that radiological progression would be result in increased symptoms, such that increased symptoms should trigger MRI scanning. However, in many cases, radiological progression does not correlate with progression of symptoms. After radiological progression, increased frequency of surveillance could be useful to monitor arthrosis (consensus level: moderate).

Any model of symptom-based, imaging-based, or self-care-based surveillance requires in-built simple re-access to surgical specialists and MDT review upon symptomatic relapse, such as in most patient-initiated follow-up (PIFU) programmes.

### Surveillance during and after systemic therapies

For patients on systemic therapy, monitoring treatment response is often based on clinical improvement in symptoms and radiological response (consensus level: strong). Variable surveillance intervals were discussed (e.g. three or six months after initiating systemic therapy), indicating that clinical context influences imaging frequency. More frequent imaging (e.g. every three to four months) could be warranted for patients early in active systemic therapy, but frequency of imaging for treatment response could be reduced (e.g. to every six months) based on duration and durability of treatment (consensus level: moderate).

## Discussion

Experts identified a complex interplay of system-level, clinical, and perceptual factors driving variation in management of TGCT across the UK. Thus, consensus statements were developed for alignment (Table I). Key issues include limited awareness of systemic therapies, regional resource constraints, reimbursement policies focused on cancer therapy outcomes, such as survival, rather than more appropriate outcomes for TGCT such as quality of life, and the fact that TGCT is often managed outside dedicated specialist centres (usually sarcoma centres). These have led to fragmented access to and administration of systemic treatments. Additionally, there is an unmet need for a holistic approach to pain and function in patients with TGCT, as there are no pain specific or physiotherapy protocols.^11^ However, regional differences, strict referral criteria (e.g. first line therapy have failed), and high demand may preclude these coming to fruition.

**Table I.** Consensus level for recommendations.

Statement/recommendation	Consensus level
**Nomenclature and characterization**	
The field is in a transitional phase, with TGCT emerging as the preferred nomenclature in oncology and multidisciplinary discussions.	Moderate
Subtyping of TGCT should be determined by radiological appearance, rather than histopathology.	Strong
Any well-circumscribed focal mass(es), without infiltrative growth patterns, is classified as L-TGCT.	Moderate
The possibility of an intermediate or multifocal localized category, especially when more than one well-defined lesion is present in different joint compartments but without the features of D-TGCT (i.e., infiltration) was proposed.	Low
**Diagnostics**	
MRI is the optimal imaging modality for diagnostic identification and characterization of TGCT. MRI should be recommended as part of the standard diagnostic work-up.	Strong
Conventional radiography has limited value but may identify calcifications, bone erosions, subchondral cysts, and joint degeneration, particularly in in longstanding or advanced disease.	Strong
Contrast agents may not be necessary and may be resource dependent (as non-contrast scans free up local imaging capacity).	Low
For TGCTs where high-quality MRI are interpreted by musculoskeletal radiologists with extensive experience of assessing TGCT and the MRI is highly suggestive (e.g., suspected D-TGCT displaying blooming artifacts), core-needle biopsy was deemed unnecessary.	Strong
For small, localized lesions, en bloc excisional biopsies could proceed directly if the radiological features are consistent with that of TGCT.	Moderate
For localized lesions, tissue-confirmed diagnosis via core-needle biopsy before excision may be appropriate in some cases where imaging is inconsistent with TGCT after review in a sarcoma MDT.	Low
All removed surgical specimen should be sent for histopathological confirmation of diagnosis. If the radiological characteristics are atypical, then the case should be referred to the local sarcoma centre as per national UK guidelines to rule out a differential diagnosis of sarcoma.	Strong
Biopsies for large, diffuse, recurrent TGCT or atypical radiological appearance should be reviewed at a sarcoma centre and/or with experienced musculoskeletal histopathologists	Strong
There is strong support for pathology review by a specialist sarcoma pathologist and access to additional diagnostic tests.	Strong
**Principles of treatment**	
Patients with suspected D-TGCT, TGCT in atypical locations, or those requiring complex procedures be reviewed through a centralized MDT case review at a sarcoma centre, given the multidisciplinary nature of TGCT management.	Strong
**Surgical techniques**	
The primary approach for asymptomatic L-TGCT should not be surgical. Active surveillance is recommended in the absence of symptoms or functional impairment.	Strong
Excisional biopsy or macroscopic complete resection should be universally recommended for symptomatic L-TGCT.	Strong
The use of arthroscopic resection could be appropriate for lesions that can be removed en bloc without contamination of the joint at easily accessible joint locations, particularly in anterior compartments or in specific joints (e.g. knee or ankle).	Moderate
Open approach was recommended for symptomatic L-TGCT due to concerns about piecemeal removal during arthroscopy.	Moderate
Posterior arthroscopies of the knee and ankle should not be advised due to technical difficulties achieving complete macroscopic resection and concerns with contaminating the joint.	Strong
The primary approach for asymptomatic D-TGCT should be active surveillance with regular clinical follow-up to assess disease progression and/or symptom development.	Moderate
MDT review and discussion, including surgical, medical, and clinical oncology, and radiology teams, should be recommended for treatment planning.	Strong
Symptomatic D-TGCT should be managed by a multidisciplinary sarcoma centre and MDT review and discussion, including surgical, medical/clinical oncology, and radiology teams, should be recommended for treatment planning	Strong
Complete macroscopic resection is the preferred approach if achievable with acceptable morbidity and preservation of function.	Strong
Arthroscopic surgery is not favored for D-TGCT due to the risk of widespread disease dissemination.	Strong
If complete resection is not feasible, partial resection (debulking) surgery of the symptomatic portion of the disease (e.g., anterior compartment of knee) should only proceed if recommended by the sarcoma MDT review.	Moderate
Experts agreed that debulking is a practical but often a suboptimal option that is performed due to lack of better alternatives which should be discussed with a sarcoma MDT.	Moderate
Arthroplasty (joint reconstruction or arthroplasty) may be appropriate in TGCT patients but only when secondary degenerative joint disease (e.g. osteoarthritis or joint erosion) is advanced and mobility is not preserved. Arthroplasty is not used as a treatment for TGCT directly and does not reduce recurrence risks.	Strong
Decisions for referrals are made on a case-by-case basis within an MDT setting.	Strong
Several surgical experts suggested a proactive approach, referring to oncology early in the planning process if disease is diffuse, only partially resectable, recurrent, or in difficult surgical locations (e.g., posterior capsule or ankle).	Low
**Radiotherapy**	
Radiation as a treatment for TGCT should be limited to only very select patients after sarcoma MDT review and only once all other options are exhausted.	Strong
**Systemic therapy**	
Referral for consideration of systemic therapies is recommended for discussion of alternatives for patients with recurrent TGCT, or D-TGCT or L-TGCT in complex locations.	Moderate
Systemic therapy may be preferred following MDT discussion and patient preference.	Moderate
Medical oncology referral is usually not necessary in the initial treatment settings, but is recommended early in cases of symptomatic, diffuse, recurrent, or unresectable TGCT, or when surgery is associated with partial resection and high morbidity, or when surgical options are exhausted, carry a high risk of local recurrence is associated, or involve difficult surgical locations (e.g., posterior capsule or ankle).	Moderate
Symptomatic recurrence may be a common trigger for medical oncology involvement.	Low
imatinib is the standard-of-care systemic therapy option in the UK at present. However, clinical trials should be the preferred option when available.	Strong
There could be a role for neoadjuvant therapy, especially in cases of bulky disease or when systemic therapy may improve surgical outcomes.	Moderate
**Surveillance and follow-up**	
Regular postoperative imaging or routine imaging was unnecessary for patients with L-TGCT even with marginal excision and that follow-up imaging should be based on symptoms.	Strong
Clinical symptoms and physical function should be used to guide post-operation decision-making. Flexible, symptom-guided imaging was recommended by all experts.	Strong
After radiological progression, increased frequency of surveillance is useful to monitor arthrosis.	Moderate
Postoperative baseline imaging was recommended for patients with D-TGCT to assess residual disease.	Moderate
Follow-up and routine imaging thereafter should be guided by patient’s symptoms and evaluated at six and 12 month intervals.	Strong
If postoperative or routine imaging is sought, MRI is unanimously preferred by all experts as the imaging modality of choice for evaluating the condition.	Strong
For patients on systemic therapy, monitoring treatment response is often based on clinical improvement in symptoms and radiological response.	Strong

D-TGCT, diffuse tenosynovial giant cell tumour; L-TGCT, localized tenosynovial giant cell tumour; MDT, multidisciplinary team; TGCT, tenosynovial giant cell tumour.

This study has limitations inherent to Delphi methodology, including reliance on expert opinion, panel selection bias, and the use of predefined thresholds to define consensus. Although structured feedback encourages convergence of views, it may also limit the expression of other perspectives. While we aimed to include experts from independent, geographically diverse, high-volume TGCT centres, not all relevant experts or centres may have been represented, and the findings may reflect regional practice patterns. These recommendations represent clinical perspectives and opinions rather than high-level evidence and should not replace prospective clinical research. In addition, the evidence base in TGCT remains limited and evolving, so conclusions may change as new data emerge. Finally, full anonymity was not preserved due to 1:1 outreach by the facilitator, which may have introduced bias.

Experts expressed unanimously that increasing awareness of, and providing education to, UK orthopaedic and medical/clinical oncologists is critical. This includes presenting data on available therapies (e.g. CSF1R inhibitors), educating that TGCT while a benign disease, has serious consequences for patients impacting function and quality of life, clarifying that a range of effective treatment options exist, and providing access to new treatments while emphasizing unmet needs. These goals are best achieved in collaboration with patient advocacy groups (e.g., TGCT Support) and clinician societies (e.g. British Orthopaedic Oncology Society, British Sarcoma Group). Protocol sharing and building structural support across the NHS via expanded access through hub-and-spoke models.

**Take home message**

- This study provides clinically important consensus guidance for managing tenosynovial giant cell tumours (TGCTs) by standardizing definitions of disease subtypes and clarifying what constitutes unresectable disease.

- It establishes clear referral criteria to specialist sarcoma multidisciplinary teams, ensuring patients with complex, diffuse, or recurrent TGCTs receive multidisciplinary evaluation and access to advanced treatments.

- Overall, this consensus guidance promotes more consistent, equitable, and multidisciplinary care across the UK.

## Data Availability

All data generated or analyzed during this study are included in the published article and/or in the supplementary material.
